# Comparison of torque expression in esthetic brackets

**DOI:** 10.4317/jced.56102

**Published:** 2019-09-01

**Authors:** Karine Martelli, Karina-Maria-Salvatore Freitas, Patrícia-de Oliveira Negreiros, Guilherme Janson, Rodrigo-Hermont Cançado, Fabricio-Pinelli Valarelli, Marcos-Roberto de Freitas

**Affiliations:** 1 DDS. M.Sc. Orthodontic Graduate student. Department of Orthodontics. UNINGA University Center, Brazil; 2 DDS., M.Sc., Ph.D. Professor. Department of Orthodontics. UNINGA University Center, Brazil; 3 DDS., Orthodontic Graduate Student. Department of Orthodontics. Bauru Dental School. University of São Paulo, Brazil; 4 DDS., M.Sc., Ph.D. Professor. Department of Orthodontics. Bauru Dental School, University of São Paulo, Brazil; 5 DDS., M.Sc., Ph.D. Professor. Department of Orthodontics. UNINGA University Center, Brazil; 6 DDS., M.Sc., Ph.D. Professor. Department of Orthodontics. Bauru Dental School, University of São Paulo, Brazil

## Abstract

**Background:**

The objective of this work is to test the null hypothesis that there is no difference in the torque expression among different esthetic brackets.

**Material and Methods:**

Five ceramic self-ligating brackets (In-Ovation C – GAC, Damon Clear–Ormco, QuicKlear-Forestadent, Click-It -TP Orthodontics, Clarity SL-3M Unitek) and 4 ceramic conventional brackets (Inspire Ice–Ormco, InVu Ceramic-TP Orthodontics, Ceramic Roth–Morelli, Clarity Metal-Reinforced Ceramic Bracket-3M Unitek) were selected. Metallic Damon MX self-ligating bracket (Ormco) was used as control. Third-order moments were measured at 12º, 24º and 36º using an archwire torsion device associated with a Universal Testing Machine (EMIC DL2000), with 0.019x0.025-inch stainless steel wire. Anova followed by Tukey tests were used for intergroup comparisons.

**Results:**

In all tested angulations the Damon Clear bracket presented the highest torque expression, followed by Clarity, Clarity SL and Damon Mx brackets, with the worst torque expression shown by the InVu Ceramic bracket. The InVu Ceramic demonstrated the largest while the Damon Clear brackets demonstrated the smallest slot height and clearance.

**Conclusions:**

The null hypothesis was rejected since torque expression was different among the esthetic brackets evaluated. It was also concluded that the slot height is directly related to torque expression.

** Key words:**Torque, orthodontic brackets, orthodontic appliances, incisor, ceramics.

## Introduction

Orthodontic treatment involves three-dimensional control of the crown and during the performed movements, the buccolingual inclination of the long axis of the tooth have fundamental importance in functionally stable occlusal relationships (1). These inclinations are achieved by a moment generated by the torsion of the rectangular wire in the bracket slot, called torque (2). Torque is expressed when the slot is filled and when the arch gradually increases in size during treatment. However, a percentage of torque is lost in the clearance between the slot and the wire (3).

The expression of torque depends on the properties and dimension of the arch, dimension of the bracket slot, bracket design, and torsion degree of the arch into the bracket slot (4-9).

Self-ligating brackets have been developed in an attempt to solve such problems, justifying that elimination of metal or elastomeric ligatures used to connect the wires to the brackets improve clinical efficacy (10,11) by reducing friction (12). Self-ligating brackets may be divided into two main categories: active and passive, according to the slot closing mechanisms. Active self-ligating brackets have a spring that stores energy and pushes against the archwire (13,14). There are reports that any advantage in decreasing friction with active self-ligating is reduced when rectangular wires are placed (10,15) resulting in better control of torque movements, rotation and inclination of teeth (11). Active self-ligating brackets seem to have better control of torque. It is a direct result of its active clip that forces the wire into the bracket slot (3).

Passive self-ligating brackets usually have a sliding flip that can be closed without invading the lumen of the slot, not exerting active forces in the arch and creating a clearance with the bracket after closing (13,14).

Ceramic brackets are designed to improve esthetics during orthodontic treatment (16,17). Fixed orthodontic brackets that combine esthetics and good technical performance are very desirable. Nowadays, there are many adults searching for orthodontic treatment, especially women claiming for esthetic appearance to accept the braces (18,19).

Esthetic brackets are delicate, so they are more prone to fractures during the torque and inclination movements (20-22). However, it seems that actually, even with greater fracture possibilities of ceramic brackets during torsion of the archwires, this kind of bracket seems to be appropriate for clinical use (20).

In the literature there are few studies that compare the expression of torque between esthetic brackets. This study aimed to evaluate the torque expression in different types of esthetic, conventional and self-ligating brackets, by comparing them with the Damon MX metal bracket. The null hypothesis was tested that there is no difference in the torque expression among different esthetic brackets.

## Material and Methods

Ten types of brackets from six different brands were used with 50 maxillary right central incisor brackets, with a slot size of 0.022-inch. Five ceramic self-ligating brackets (In-Ovation C – GAC, Damon Clear–Ormco, QuicKlear-Forestadent, Click-It -TP Orthodontics, Clarity SL-3M Unitek) and 4 ceramic conventional brackets (Inspire Ice–Ormco, InVu Ceramic-TP Orthodontics, Ceramic Roth–Morelli, Clarity Metal-Reinforced Ceramic Bracket-3M Unitek) were selected. Metallic Damon MX self-ligating bracket (Ormco) was used as control (9). Five of each were tested. For the tests, 50 stainless steel wire segments (TP Orthodontics, La Porte, Indiana, USA), 3.5 cm long, with rectangular section of 0.019x0.025-inch were used (Batch 10713087SMS). The following materials were used: Orthodontic Sticker; Orthodontic resin; Red Orthodontic Elastic Modules for ligation (Batch 1701799).

A universal testing machine EMIC DL2000 (Instron) was adapted to a device for wire and bracket torsion tests. The device was designed to perform symmetric wire torsion. The device has a base for adaptation of the cylinders when the brackets are bonded for testing.

**Figure 1:**
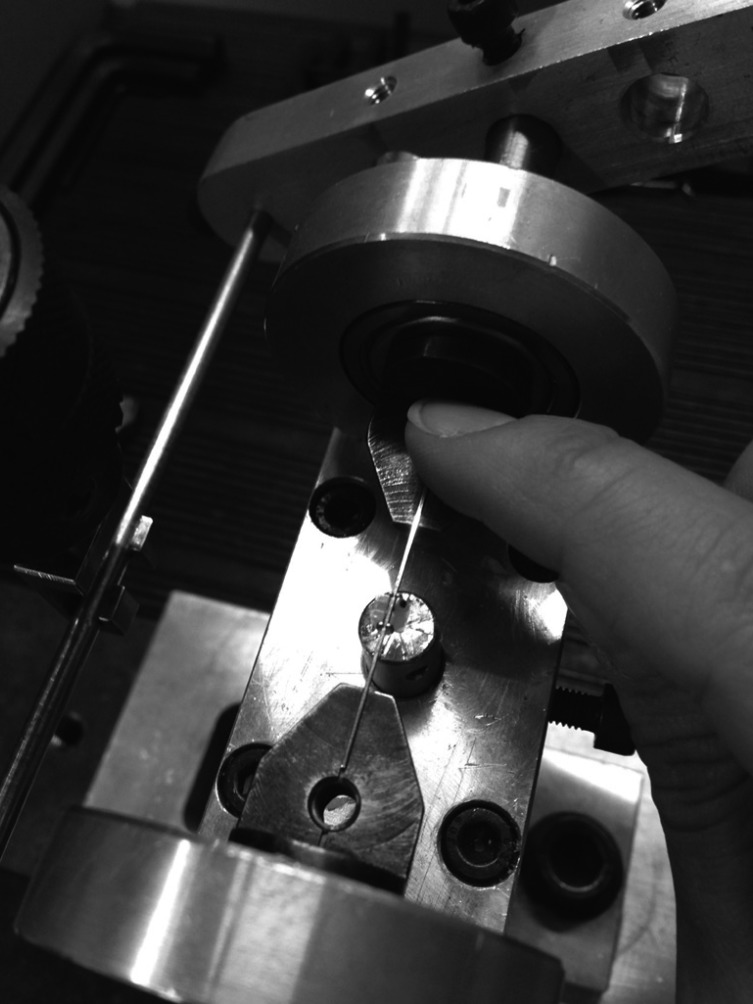
Universal testing machine with the wire-bracket system positioned for torsion test.

To fix the wire, other two stainless steel metal cylinders were manufactured, each one 4 cm in length and approximately 1 cm in diameter, in order to have accessories bonded to them. The system provides a perfect alignment and rotation of the wire around its long axis. For this, the torsion device guarantees the alignment between the wire and the bracket slot. Initially, the central cylinder is positioned and then the wire, already connected with the bracket, is positioned (Fig. 1).

For bonding, orthodontic adhesive was applied in the bracket and cylinder base with applicators. After this, curing laser light was applied for 20 seconds. Then, the orthodontic resin was applied to bond the base of the cylinder with the bracket already connected to the wire. Finally, curing laser light was applied for 40 seconds and the tests were performed immediately after bonding.

Prescription of torque in the brackets did not affect the study, since the zero torque position was used as an initial baseline for all tested brackets.

**Figure 2:**
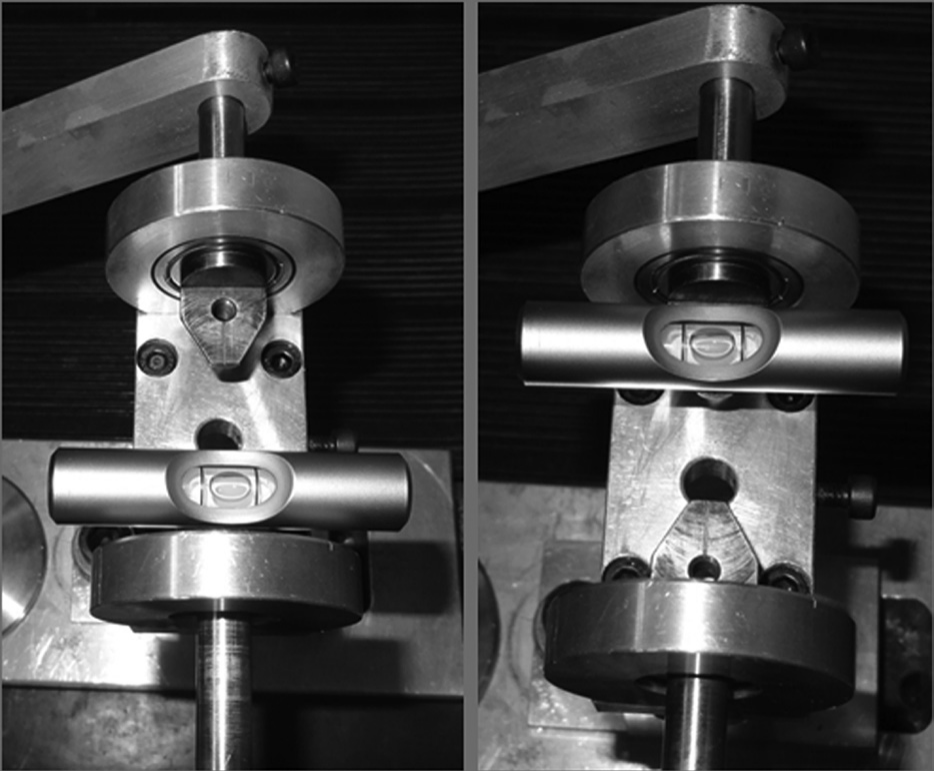
“Bubble Level” accessory to leveling the system in each start test.

**Figure 3:**
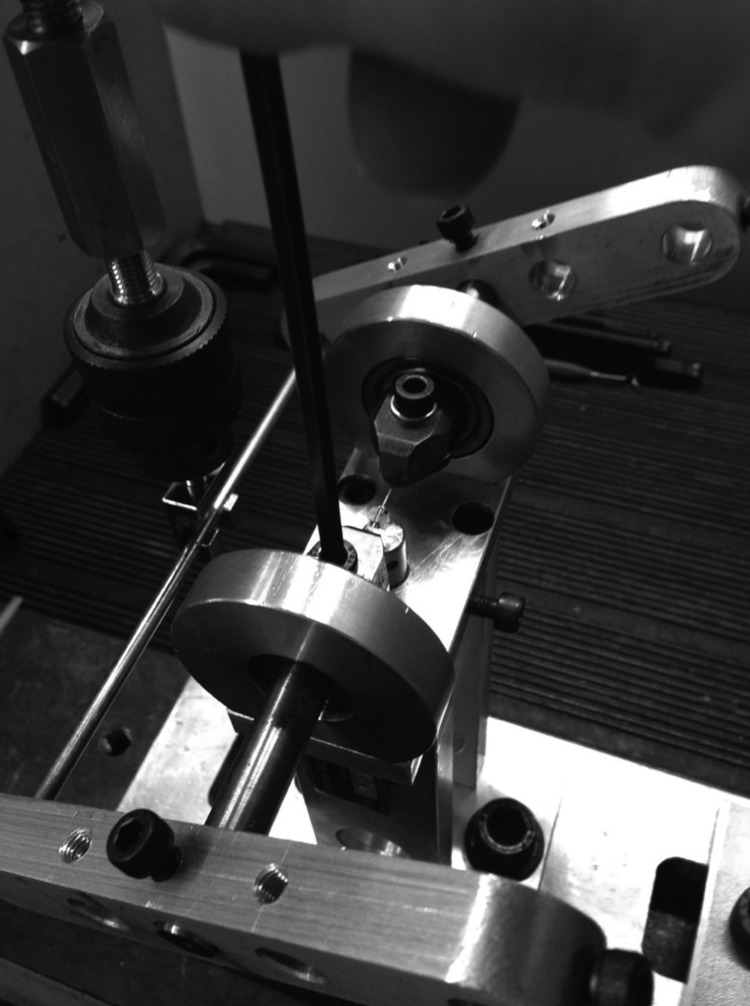
The system attached by screws to avoid slipping of the set that could impair the results.

In each test, the machine was reset in the system, and new leveling was performed in order to avoid residual forces and torques of the previous test. The leveling system was assisted by an accessory termed “bubble level” (Fig. 2). The system was attached by screws to the lateral cylinders that have a slot to guide the wire position with the bracket slot, to avoid slipping of the set that could impair the results (Fig. 3).

Each bracket/wire combination was tested once at the angles of 12º, 24º and 36º, and for each angle of the wire the force moment was measured in “Nmm” (Newton x mm) and then converted to an angle number.

The twisting device consisted of a double arm torsion which allowed conversion of the articulated rod under 0.25 rpm in movement to spin the lateral cylinders. The torque was measured by a transducer in the test with a strain gauge load cell that was used to measure the components of forces and moments (torque) of the bracket/wire combinations tested. The load cell used was the “Trd 19” 200 N.

A sensor captured the forces and torques applied, the angle of torsion, and electronically transferred the data to the computer, that could translate and display the results in graphical form. A data capturing software (Tesc version 3.01) was used to capture the transducer signal and register it to the file.

After the tests were finalized, the slot heights of 5 brackets of 10 bracket brands were measured to complement this study, using a profile projector (Starrett ® VB 300). The distance between the upper and lower slot walls of each bracket was measured. The height and width of 5 segments of wire were measured with an external micrometer (Micromaster IP54, TESA SA, Switzerland).

Clearance of the slot is characterized by the angle formed between the wire section ends with the wall of the bracket slot (5). To calculate the angle at the moment that the wire touches the upper and lower walls of the bracket slot it was necessary to measure the height of the bracket slots used in the tests and the height of the wire segments with the same dimensions and from the same batch (23). The slot clearance was calculated with the HP 50g Graphing Calculator for a 0.019 x0.025-inch stainless wire in a 0.022 x0.028-inch slot bracket.

The clinically effective range of torque angles considered by the literature is 5 to 20 Nmm (3,24,25). Therefore, the corresponding torsion angle to produce the clinically effective range of torque angles for each bracket type was calculated.

The calculation consisted in converting the deflection (mm) in angle using a rule of three calculation, where 323.5 mm is the device circumference, which equals 360°. Therefore, the amount of deflection, in mm, generates a proportional angle.

Torque moment was calculated by multiplying the force (N) by the distance (mm). This distance is the device lever arm length which is equal to 51.5 mm.

-Statistical methods

Assessment of data normality was performed with Kolmogorov-Smirnov tests, that showed normal distribution for all variables. Therefore, intergroup comparisons were conducted by one-way ANOVA followed by Tukey tests.

Descriptive statistics of the heights and clearances of all tested types of brackets and the corresponding torsion angle to produce the clinically effective range of torque angles of each bracket type were performed.

A Linear regression analysis was performed to check how the wire torsion angle is responsible for the moment of force expressed in the bracket slot. The statistical analyses were performed with Statistica software (Statistica for Windows 6.0; Statsoft, Tulsa, Oklahoma, USA). Results were considered significant at *P*<0.05.

## Results

InVU Ceramic brackets demonstrated the significantly smallest forces developed for all torque angles as compared to the Damon Clear brackets, which showed the highest forces for all torque angles. Individual comparisons for all brackets are displayed in  Table 1. QuicKlear brackets fractured before reaching 24 degrees of twist. QuicKlear, Click-it and Ceramic Roth brackets fractured before reaching 36 degrees of twist.

**Table 1:**
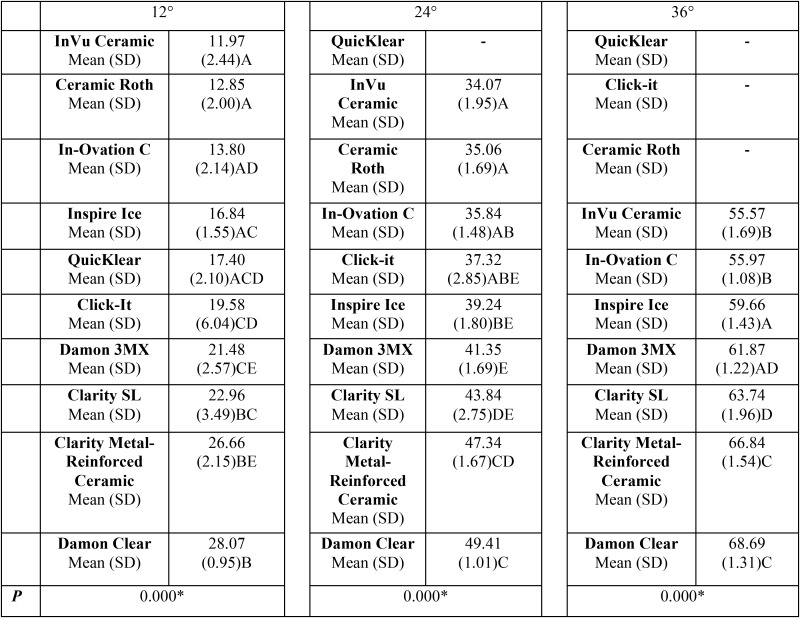
Intergroup comparison of the traction force of ten types of brackets used in three torque angles (Nmm, n=5, ANOVA followed by Tukey tests). The results are displayed in ascending values order in each torque angulation.

The Damon Clear demonstrated the smallest while the InVu Ceramic brackets demonstrated the largest slot height and clearance ( Table 2).

**Table 2:**
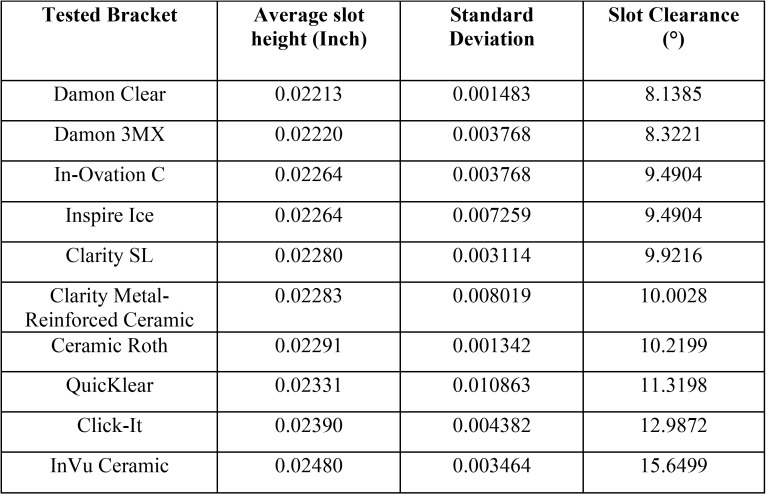
Slot heights and clearances of the different types of tested brackets with wire dimensions of 0.0188 x0.02479-inch (n = 5).

The Damon Clear demonstrated the smallest while the InVu Ceramic brackets demonstrated the largest torsion angles to produce the clinically effective range of torque angles ( Table 3).

**Table 3:**
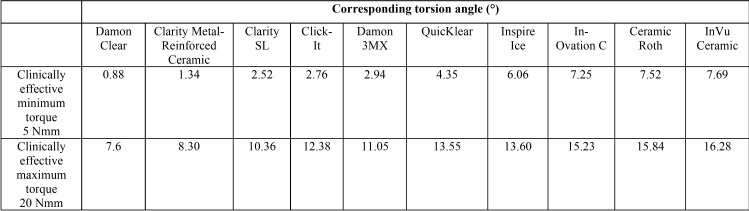
Clinically effective range of torque angles (5 to 20 Nmm).

The regression analysis indicated that 94.37% of the force moment expressed in the bracket slot can be explained by the slot height and the torsional angle in the wire. The remaining 5.63% of variation is explained by other variables as the type of slot and clip material, steel and ceramic, or the type of bracket, conventional and self-ligating brackets, or even the slot closing mechanisms, passive and active, not included in the regression model ( Table 4).

**Table 4:**
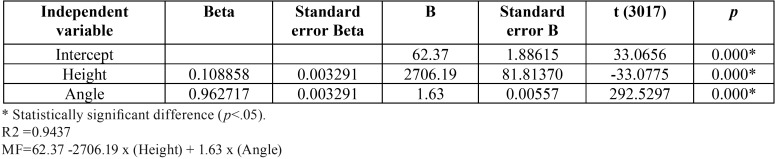
Multiple linear regression analysis results considering the Torsion Moment (MT) as the dependent variable for all evaluated brackets. Regression Summary for Dependent Variable Moment of force.

## Discussion

Significant differences were observed between the twisting forces of several brackets. InVu Ceramic demonstrated the smallest forces while the Damon Clear brackets showed the highest torque value for all angles tested ( Table 1).

These differences can be explained by the different slot heights of the brackets and consequently their slot clearance. The Damon Clear demonstrated the smallest while the InVu Ceramic brackets demonstrated the largest slot height and clearance ( Table 2). Therefore, it is quite obvious that these results would be expected. These differences in slot height also explain the corresponding torsion angles of the brackets to produce the clinically effect range of torque angles ( Table 3).

These results were also confirmed by the regression analysis that demonstrated that the force moment expressed in the bracket slot can be explained by the slot height and the torsional angle in the wire ( Table 4). Another study confirms the results obtained (26).

According to the DIN 13971-2 norm introduced in 2000 to regulate the nominal dimensions of the brackets and their tolerance limits, the height of the bracket slot was 0.022-inch, varying from 0.559 mm (which corresponds exactly to 0.022-inch) up to a maximum of 0.599 mm (which corresponds to 0.023-inch). In this research, the height of all slots was above the minimum tolerance limit recommended by the norm. The bracket closest to the minimum limit was the Damon Clear, with 0.562 mm (0.02213-inch). Damon 3MX, In-Ovation C, Inspire Ice, Clarity SL, Clarity, Ceramic Roth and QuicKlear showed intermediary slot sizes, inside the tolerance limits. Click-it and InVu Ceramic brackets showed slot heights above the maximum recommended limit.

Different ligation mechanisms were tested: conventional brackets with elastomeric ligatures and active and passive self-ligating brackets. The results showed that behavior of the brackets tested was independent of the ligation mechanisms that each presented.

Another work that also supports the findings of this study evaluated whether the clip of active brackets interfered in the final torque expression. At the end, there was no significant difference between the two systems, which demonstrated that it does not matter if the bracket is active or passive. What really matters in the transmission of torque is the dimension of the bracket slot (27). Nevertheless, there was one investigation that showed that active ligating brackets are more effective in expression of torque (3). Probably, the different result in this study was consequent to the difference in slot height of the tested brackets.

The tests also showed that the material of the clip did not affect the results. Regarding the material of the slot, the results were in general also independent of the presence or not of slot metal reinforcement. Metal slot reinforcement of the ceramic bracket is similar to metal brackets regarding friction (26). The current results showed that they are also similar regarding torque.

Dimension of the slot seem to be the most important factor that affects torque expression. In several studies the slot of the brackets presented with the dimensions above that reported by the manufacturer. Consequently, clearance between the wire and the slot increases and will negatively affect the torque expression (28,29).

Therefore, the results suggest that any attempt in comparing the influence of bracket ligating system on torque expression should initially compare the slot dimensions. Reliable comparisons cannot be performed if the bracket slots have different dimensions.

Torque is responsible for the buccolingual movement of the root and crown of teeth, and this is very important in orthodontic mechanics, to establish a good final occlusion. Since torque expression varies in different brackets, orthodontists should know which brackets express more or less torque, and use this information during treatment mechanics. This way, a clinically effective torque can be used in conjunction with the bracket cho¬sen by the orthodontist.

## Conclusions

• The null hypothesis was rejected since torque expression was different among the esthetic brackets evaluated.

• The InVu Ceramic bracket showed the smallest torque expression and the Damon Clear bracket showed the largest torque expression in all tested angles;

• The InVu Ceramic demonstrated the largest while the Damon Clear brackets demonstrated the smallest slot height and clearance;

• Therefore, it was concluded that the slot height is directly related to torque expression.
